# Bridging the gap in gastrointestinal healthcare in a resource-limited setup: Feasibility study of weekend endoscopy services in Southwest Ethiopia

**DOI:** 10.1055/a-2625-6225

**Published:** 2025-07-01

**Authors:** Guda Merdassa Roro, Elias Merdassa Roro, Tsegaye Melaku, Esayas Kebede Gudina

**Affiliations:** 1 37602 Gastroenterology and Hepatology Unit, Department of Internal Medicine, School of Medicine, Addis Ababa University, Addis Ababa, Ethiopia; 2 128159 Department of Public Health, Institute of health sciences, Wollega University, Nekemte, Ethiopia; 3 420004 Mater Research Institute, The University of Queensland Faculty of Medicine, Brisbane, Australia; 4 661018 School of Pharmacy, Jimma University Institute of Health, Jimma, Ethiopia; 5 661018 Department of Internal Medicine and Jimma University Clinical Trial Unit, Jimma University Institute of Health, Jimma, Ethiopia

**Keywords:** Other focus (of reviewers), Epidemiology, Endoscopy Lower GI Tract, Endoscopy Upper GI Tract

## Abstract

**Background and study aims:**

Endoscopy is essential for diagnosis and management of gastrointestinal disorders. However, its accessibility in Africa is limited by the need for extensive training and costly equipment. This study aimed to assess the feasibility of a weekend outreach endoscopy service led by a trained gastroenterologist in southwest Ethiopia, where endoscopy services were previously unavailable.

**Patients and methods:**

A weekend outreach endoscopy service was launched in 2019 at a primary hospital in Jimma City, located 360 km from Addis Ababa. Procedures were performed using the Fujinon EPX-2500-HD system. Demographic data, endoscopy findings, and histology results were documented electronically. Findings were compared with those from four Ethiopian referral hospitals offering full-time endoscopy services.

**Results:**

A total of 2165 esophagogastroduodenoscopies (EGDs) were performed with a diagnostic yield of 93.3%. The most common indications for EGD were dyspepsia (53.7%) and dysphagia (17.0%). Patients who underwent endoscopy for alarm symptoms as an indication had a 77% to 83% chance of having a major finding compared with those with dyspepsia without an alarm symptom (24%). Squamous cell carcinoma (40.2%), adenocarcinoma (29.6%), and chronic nonspecific inflammation (16.2%) were the predominant histologic findings among those who had a biopsy (n = 425).

**Conclusions:**

The study demonstrates the feasibility and effectiveness of a weekend outreach endoscopy service led by a trained gastroenterologist in a rural Ethiopian setting. The unexpectedly high prevalence of upper gastrointestinal disorders, including cancers, and the long duration of symptoms before endoscopy likely reflect delayed diagnoses due to limited access to endoscopy. Moreover, presence of alarm symptoms predicted major endoscopic findings. Expanding endoscopy services, increasing public awareness, and further research into risk factors and preventive strategies for these diseases are recommended.

## Introduction

Gastrointestinal diseases represent a significant health burden worldwide, with upper gastrointestinal conditions such as peptic ulcers, gastritis, and esophageal cancer contributing substantially to morbidity and mortality 1 . Esophagogastroduodenoscopy (EGD) is the gold-standard test for investigation of upper gastrointestinal symptoms, allowing direct mucosal visualization, tissue acquisition and, when required, therapeutic interventions 2 . The diagnostic capabilities of EGD improve clinical outcomes while reducing healthcare costs and resource utilization, establishing it as an essential service in many healthcare systems worldwide 3 .

Despite the proven benefits of endoscopy, its accessibility in low-resource settings remains limited, particularly in sub-Saharan Africa 4 5 . Endoscopy capacity in eastern sub-Saharan Africa, including Ethiopia, is severely limited, ranging from just 1% to 10% of that reported in resource-rich countries, despite a high burden of gastrointestinal diseases 6 .

Barriers to development of endoscopy services include a shortage of endoscopists with advanced training, as well as a lack of equipment and basic infrastructure 5 . Establishing safe and effective endoscopy service requires extensive training, investment in expensive equipment and accessories, and adequate disinfection facilities 4 7 . Endoscopists need to receive comprehensive training in both the technical skills of endoscopy and the cognitive understanding of gastrointestinal conditions to interpret findings and develop effective patient care plans accurately 7 8 . Many organizations, such as the World Gastroenterology Organization (WGO), the European Society of Gastrointestinal Endoscopy (ESGE), and the World Endoscopy Organization (WEO), have implemented various strategies, including training partnerships and material support, to address these gaps 4 5 .

In Ethiopia, most endoscopic services are concentrated in major cities and referral hospitals, leaving rural populations underserved 9 . For instance, as of 2018, there was no functional endoscopy service across the entire West and southwest Ethiopia, a region home to over 22 million people. To address this significant service gap, we identified Jimma City as an ideal location to reach at least half of the population in these underserved regions by establishing a weekend outreach program for upper gastrointestinal endoscopy and colonoscopy services. To accelerate these efforts, doctors working at Jimma University Hospital facilitated launch of a collaborative weekend outreach endoscopy program between a gastroenterologist from Addis Ababa University College of Health Sciences (AAU-CHS) and Jimma Awetu Hospital, a private facility in Jimma City.

This study aimed to evaluate feasibility and diagnostic yield of a weekend outreach endoscopy program in predominantly rural communities in southwest Ethiopia. Specifically, it sought to compare clinical, endoscopic, and histologic findings among patients referred from regions without endoscopy services to those from hospitals with established, full-time onsite endoscopy services in major Ethiopian cities. By assessing effectiveness and impact of this outreach initiative, the research highlighted the healthcare needs of rural populations and advocated for enhanced endoscopy services in these and similar underserved areas.

## Methods

### Study settings and participants

In early 2019, an endoscopy service was established at Jimma Awetu Hospital, a private facility located in Jimma City, approximately 360 km from Addis Ababa, Ethiopia. This weekend outreach program was launched in collaboration with a senior gastroenterologist from the AAU-CHS, who travels to Jimma every 2 weeks and aimed to serve the gastrointestinal diagnostic needs of underserved populations in southwest Ethiopia. Eight nurses at the hospital were trained to handle patient scheduling, procedure preparations, and equipment disinfection and assist with premedication, documentation, and biopsy sample management.

Patients from the surrounding areas were referred by healthcare providers from government and private facilities that were informed of program availability. The service provided included diagnostic upper endoscopy, colonoscopy, biopsy collection, and comprehensive reporting on endoscopic findings, endoscopic diagnosis, histopathology diagnosis, and detailed treatment recommendations. Patients or their guardians provided written informed consent for conscious sedation, as needed, and to undergo endoscopy, following an explanation of risks and benefits.

### Procedure details

The procedures were performed using a Fujinon EPX-2500-HD system from Fujifilms in a dedicated three-room endoscopy unit within the hospital. The patients were advised to fast from solid food for 8 hours and from fluids for 2 hours. A brief history was taken to confirm the appropriate indication for the procedure 7 . Topical anesthetic spray or low-dose intravenous diazepam (5–7.5 mg) was used as premedication, with only a few patients requiring sedation with propofol. Patients were positioned in the left lateral decubitus position and the examination adhered to standard endoscopic guidelines 10 11 . Based on indications, biopsy samples were collected, preserved in formalin solution, and sent for histopathology examinations 12 .

### Data management and analysis

Endoscopy reports were documented electronically and stored on a desktop computer in the endoscopy unit. Demographic and procedure-related information was entered into Microsoft Excel 2019 immediately after each procedure. Histopathology results were updated upon receipt, typically during subsequent weekend sessions. All data, including patient demographics, endoscopic findings, and histologic diagnoses, were then imported into IBM SPSS (version 29) for statistical analysis. Descriptive statistics were used to analyze the data, and the findings were presented using tables and graphs.

### Feasibility and diagnostic yield assessment

Program feasibility was assessed based on successful establishment and maintenance of the service, acceptance among local healthcare providers, and referral rates. Diagnostic yield was calculated as percentage of patients with at least one abnormal finding from the total EGD procedures performed. Referral sources were analyzed to assess types of healthcare institutions (hospitals or clinics) utilizing the service and to evaluate their capacity for acting on diagnostic findings.

### Ethical considerations

The study protocol was reviewed and approved by the Institutional Review Board of Jimma University Institute of Health (Ref No. JUIH/IRB/359/23). Written informed consent for the procedure was obtained following the standard hospital protocol, as detailed above. For this report, all individual patient identifiers were removed, and only anonymized data were used for analysis. Hard copies of the endoscopy and histopathology reports were provided to patients for presentation to their referring health professionals for proper management.

## Results

### Patient demographics and referral sources

**Fig. 1:**
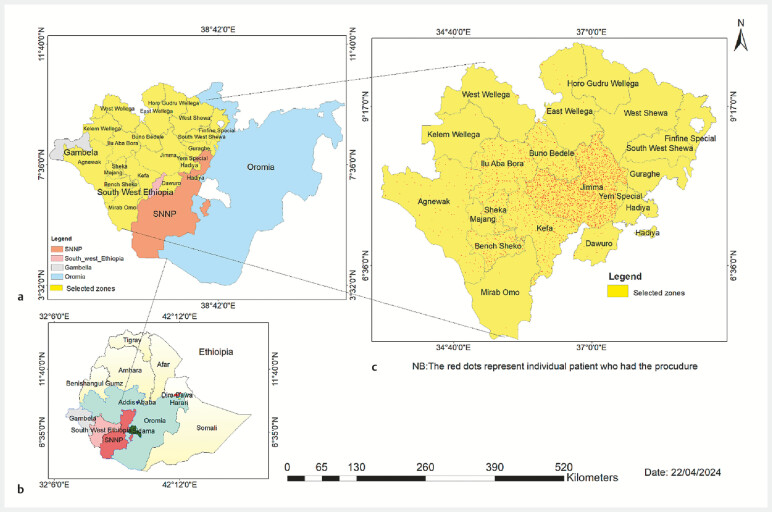
Geographic distribution of patients attending outreach endoscopy services at Jimma Awetu Hospital. **a** Regions of Ethiopia. **b** Zonal-level residential addresses of patients. **c** Density of patients from each zone receiving endoscopy services, 2018–2022.

A total of 2,165 upper gastrointestinal endoscopy (EGD) procedures were performed from October 2018 to March 2023. Median patient age was 40 years (interquartile range [IQR] 28–50), and 1214 (56.1%) of the patients were male. Patients were referred from 37 healthcare facilities, including 15 hospitals. Most patients (1688; 78%), came from 10 different zones within the Oromia Region, whereas others were referred from the Southwest/South Ethiopia People’s Region (371; 17.2%) and the Gambella Region (67; 3.1%) ( Fig. 1 ).

### Indications for esophagogastroduodenoscopy

The most common indications for the procedure were dyspepsia (1162; 53.7%), dysphagia (369; 17.0%), and epigastric and retrosternal pain (294; 13.6%). Median duration of symptoms before referral was 5 months (IQR 3–12) ( Table 1 ).

**Table:** **Table 1** Patient characteristics, referral sources, and indications for endoscopy among patients attending outreach upper gastrointestinal endoscopy services at Jimma Awetu Hospital 2018–2023.

Characteristics		Frequency	Percentage
Age category in years (n = 2165)	< 20	117	5.4
	20–29	495	22.9
	30–39	443	20.5
	40–49	436	20.1
	50–59	325	15.0
	≥ 60	349	16.1
Gender (n = 2165)	Male	1214	56.1
	Female	951	43.9
Source of referral (n = 2165)	Hospitals	2036	94.0
	Clinic	110	5.1
	Not documented	19	0.9
Indication for EGD (n = 2165)	Dyspepsia	1162	53.7
	Dysphagia	369	17.0
	Epigastric and/or retrosternal pain	294	13.6
	Vomiting	181	8.4
	Upper gastrointestinal bleeding	134	6.2
	Other	25	1.2
EGD, esophagogastroduodenoscopy.			

### Diagnostic yield and endoscopic findings

About 2020 patients (93.3%) who underwent EGD had at least one abnormality. Endoscopic features of inflammation, such as erythema and/or edema (including duodenitis, gastritis, and/or gastroduodenitis), accounted for 722 (33.3%) of the findings, followed by GERD-related esophagitis (422; 19.5%) and upper gastrointestinal masses (372; 17.2%). Among 425 documented histopathology results, squamous cell carcinoma in 171 (40.2%), adenocarcinoma in 126 (29.6%), and chronic nonspecific inflammation in 69 (16.0%) were the most frequent findings ( Table 2 ).

**Table:** **Table 2** Endoscopic and histologic findings of patients attending outreach upper gastrointestinal endoscopy services at Jimma Awetu Hospital 2018–2023.

Characteristics		Frequency	Percentage
Endoscopy findings (diagnosis) (n = 2165)	Endoscopic features of inflammation	722	33.3
	GERD-related esophagitis	422	19.5
	Upper gastrointestinal masses	372	17.2
	Ulcer or erosion	225	10.4
	Chronic ulcer complications	148	6.8
	Normal	145	6.7
	Esophageal varices	59	2.7
	Others*	72	3.3
Histological findings (n = 425)	Squamous cell carcinoma	171	40.2
	Adenocarcinoma	126	29.6
	Chronic non-specific inflammation	69	16.2
	Premalignant conditions ^†^	24	5.6
	Other benign conditions ^‡^	26	6.1
	Other malignant histology ^§^	9	2.1
GERD, gastroesophageal reflux disease. *Achalasia, nodule, polyp, papilloma, Zenker’s diverticula, esophageal candidiasis, hiatal hernia, inlet patch, and melanosis, vocal cord paralysis. ^†^ Includes chronic atrophic or active gastritis with intestinal metaplasia, Barrette esophagus, squamous cell dysplasia. ^‡^ Include polyps, unremarkable, Celiac disease, Crohn’s disease, benign ulcer. ^§^ Includes poorly differentiated carcinoma, MALT (mucosa-associated Lymphoid tumor), gastric High-grade NHL (non-Hodgkin lymphoma), GIST (gastrointestinal stromal tumor).			

### Classification of endoscopic findings and distribution of major findings according to clinical presentation and demographic characteristics

To further evaluate whether the high diagnostic yield had prognostic implications and whether traditional alarm features are associated with identification of major lesions, we broadly classified the endoscopic findings into major, minor, and normal findings 13 Accordingly, major endoscopic findings accounted for 968 (44.7%), minor findings (mild lesions) 1055 (48.7%), and normal findings 142 (6.5%) ( Table 3 ). When patients were stratified according to indication for endoscopy, major endoscopic findings were found to be very common among patients presenting with persistent vomiting (159 of 181; 88%), dysphagia (307 of 369; 83%), and upper gastrointestinal bleeding or anemia (103 of 134; 77%), respectively. In contrast, 818 of 1162 patients (70%) undergoing endoscopy for an indication of dyspepsia and 149 of 294 (51%) of those with an indication of epigastric or retrosternal pain had minor (mild) lesions on endoscopy.

**Table:** **Table 3** Classification of primary endoscopic findings in patients attending outreach upper gastrointestinal endoscopy services at Jimma Awetu Hospital 2018–2023.

Classifications	Frequency	Percentage
Major endoscopic findings		
Cancer	371	17.1
Ulcer and ulcer complications	371	17.1
Severe inflammation	81	3.7
Portal hypertension	59	2.7
Stricture(stenosis)	39	1.8
Severe GERD	26	1.2
Others (major)	21	1.0
Total major findings	968	44.7
Minor endoscopic findings		
Mild inflammation	644	29.7
Mild GERD	386	17.8
Other minor findings	25	1.2
Normal	142	6.6
Total minor (mild) and normal findings	1197	53.3
GERD, gastroesophageal reflux disease.		

Major findings were more common among males (614 of 1214; 50.6%) compared with females (354 of 951; 37.2%), with cancers more common among females (186 of 951; 19.6%) vs males (185 of 1214; 15.2%). Ulcers and portal hypertension were more common in males, 22.5% and 4.3% vs 10.3% and 0.7% in females, respectively.

Sixty-two percent of patients older than age 50 years had major endoscopic findings, with cancers accounting for 35%, which was almost threefold compared with the age group 50 and below (11.6%). Prevalence of ulcer diseases was similar, about 17% in both age categories.

The chi-squared test further demonstrated a significant difference among the groups at X ^2^ = 38 ( *P* < 0.001) for gender, X ^2^ = 126 for age ( *P* < 0.001), and X ^2^ = 596 for indications ( *P* < 0.001) ( Table 4 ).

**Table:** **Table 4** Prevalence of major and minor endoscopy findings by age and gender of patients attending outreach upper gastrointestinal endoscopy services at Jimma Awetu Hospital 2018–2023.

Characteristics		Endoscopy diagnosis classification		X ^2^	*P* value
		Major	Minor		
Gender	Male	614 (63.4%)	600 (50.1%)	X ^2^ = 38	0.001
	Female	354 (36.6%)	597 (49.9%)		
Age group	< 20	33 (3.4%)	84 (7.0%)	X ^2^ = 126	0.001
	20–29	152 (15.7%)	343 (28.7%)		
	30–39	176(18.2%)	267 (22.3%)		
	40–49	207 (21.4%)	229 (19.1%)		
	50–59	172 (17.8%)	153 (12.8%)		
	≥ 60	228 (23.6%)	121 (10.1%)		
Indications	Dyspepsia	277 (28.8%)	818 (77.1%)	X ^2^ = 596	0.001
	Dysphagia (odynophagia)	307(31.9%)	41 (3.9%)		
	Epigastric pain	109 (11.3%)	149(14.0%)		
	Upper gastrointestinal bleeding	103 (10.7%)	25(2.4%)		
	Vomiting	159 (16.5%)	13 (1.2%)		
	Other	8 (0.8%)	15 (1.4%)		

### Trends in patient referral flow and associated challenges

During the initial stage of the service, we saw only six to 30 patients per weekend visit. This was partly due to the limited scale of advertising, but a significant factor was lack of awareness about gastrointestinal endoscopy. Most physicians had no prior exposure to endoscopy procedures, because there were no such services even in most medical schools. Health professionals, patients, and their attendants were observed to lack awareness about procedure safety and diagnostic accuracy.

With increased awareness and education, both physicians and patients have gained confidence in the services. Now, even general practitioners in remote clinics are referring patients. A few patients have also come from as far as South Sudan and Sudan.

**Fig. 2:**
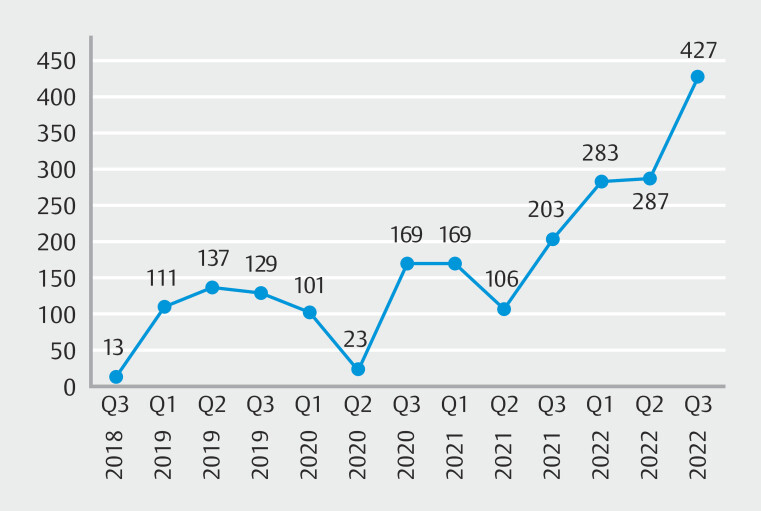
Trends in number of patients attending outreach upper gastrointestinal endoscopy service at Jimma, Awetu Hospital, Southwest Ethiopia.

The service faced several challenges. Some patients had to wait for more than 10 days until the next weekend visit to have their procedures done because we commonly run a bimonthly schedule. The COVID-19 pandemic forced a 6-month interruption ( Fig. 2 ). Frequent power outages were another significant obstacle. A major challenge was lack of onsite equipment maintenance, necessitating equipment transportation to Addis Ababa or even Dubai, United Arab Emirates, for major repairs. In addition, overlapping duty schedules at the gastroenterologist's primary station, particularly on Friday afternoons, initially posed a problem. However, this issue was resolved once the program was established and department heads at Addis Ababa University were notified. The service is now up and running with additions of endoscopic therapeutics such as esophageal variceal band ligation (EVL).

### Trends in service utilization

Of the total patients, 2,036 (94%) were referred from hospitals, whereas the remaining patients came from clinics. As awareness of the service has increased, monthly patient attendance has risen from an average of 31 in the first year to over 100 by the fourth year, supported by steady growth in the number of referring health facilities. Currently, the program performs an average of 1550 procedures annually. This analysis focused on 2,165 upper gastrointestinal endoscopies.

## Discussion

This study demonstrates the feasibility and effectiveness of a weekend outreach endoscopy service in Southwest Ethiopia, providing critical diagnostic access for upper gastrointestinal disorders in a predominantly rural population. Over 2,100 procedures were performed with a high diagnostic yield of 93.3%, highlighting the region's substantial burden of gastrointestinal diseases. Notably, the high prevalence of major upper gastrointestinal diseases in about 45%, including upper gastrointestinal malignancies (17.2%), ulcer and ulcer-related complications (17.1%), portal hypertension (2.7%) and stricture (stenotic) lesions in (1.8%), emphasizes the need for expanded endoscopic services in Ethiopia's rural areas, where diagnostic delays contribute to poor health outcomes. The number of procedures performed to diagnose one major upper gastrointestinal disease (ulcer, mass, portal hypertension, stenosis including achalasia, and severe inflammation) was 2.2.

**Fig. 3:**
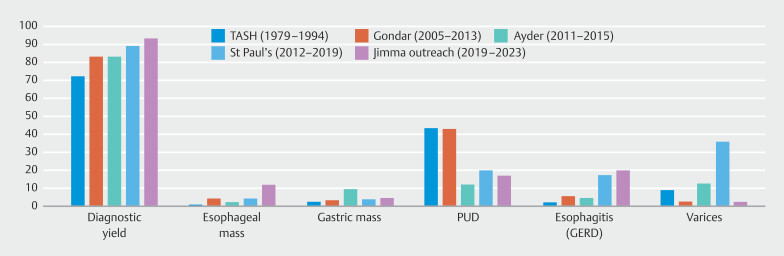
Diagnostic yield and outcomes of esophagogastroduodenoscopy as a percentage of total procedures in Jimma Awetu Hospital outreach service compared with other centers in Ethiopia.

The diagnostic yield of this outreach program exceeded that of other Ethiopian referral hospitals offering full-time endoscopy services. At the outreach site, the diagnostic yield was 93.3%, surpassing those reported from other Ethiopian centers, including St. Paul’s Hospital (89.3%) 14 , Gondar (83.4%) 15 , and Ayder (83.0%) 16 ( Fig. 3 ). One plausible reason for this difference could be the targeted nature of referrals in this outreach setting, where local healthcare providers may be more likely to refer patients with prolonged or severe symptoms due to limited availability of diagnostic services. This referral pattern could contribute to a higher prevalence of pathologic findings among patients who undergo EGD, as compared with centers with more routine access to endoscopy. Moreover, all procedures were performed by an experienced senior gastroenterologist using new endoscopy machine at the outreach site whereas gastroenterology trainees and internists with short-course onsite endoscopy training also performed procedures in the other centers 14 16 . But it should be noted that diagnostic yield is very high even in the other centers in Ethiopia ranging from 83% to 89%, likely because access is still limited and compels physicians to refer patients with likely severe organic diseases ( Fig. 3 ).

Dyspepsia remains the most common indication for upper endoscopy, accounting for 53.7% of cases in our study, consistent with previous studies from Ethiopia 14 17 . Alarm symptoms were identified in 30.1% of patients, including dysphagia (17.0%), vomiting (8.4%), upper gastrointestinal bleeding (4.0%), and anemia (0.7%). The high prevalence of alarm symptoms underscores the significant prevalence of malignancies and ulcers among our patients, highlighting the critical need for thorough evaluation of gastrointestinal symptoms in our setup.

Our findings suggest evolving trends in EGD findings in Ethiopia over the past four decades ( Fig. 3 ). Historically, duodenal ulcers were highly prevalent, detected in over 40% of approximately 10,000 EGD procedures conducted between 1979 and 1994 17 . By 2005 to 2013, this prevalence had declined to 25.4% 15 , and recent studies, including our own, show that peptic ulcer disease (PUD) has dropped to around 10% 14 16 . declining prevalence of PUD in Ethiopia over recent decades is likely due to several factors. Improved management of *Helicobacter pylori* infections, including better diagnostics and wider antibiotic use, which has led to significant decline in prevalence of *H. pylori* 18 and increased availability of proton pump inhibitors likely reduced ulcer incidence significantly.

Although rates of PUD have decreased, prevalence of patients diagnosed with esophagitis—particularly due to gastroesophageal reflux disease (GERD)—has risen significantly, from 2.3% three decades ago 17 to 19.5% in our study and St. Paul's Hospital 15% from 2012 to 2019 14 ( Fig. 3 ). This increasing prevalence of GERD-related esophagitis in Ethiopia may be linked to shifts in lifestyle and dietary habits, rising obesity rates, and specific regional factors 19 20 . In Jimma, where our study was conducted, high consumption of coffee and khat is particularly relevant 21 , and both stimulants can relax the lower esophageal sphincter and increase GERD risk 22 23 . Westernized diets higher in fats and processed foods can also exacerbate GERD symptoms, whereas a sedentary lifestyle further contributes to obesity, a known risk factor for GERD 24 . Esophageal colonization of *H. pylori* and associated esophagitis has been well described in studies and is a possible contributing factor in at least the 14% of our patients who had both gastro-duodenitis and esophagitis 25 . In addition, heightened awareness of GERD symptoms may lead to higher diagnostic rates. These combined factors, along with improved diagnostic practices, likely account for the observed increase in esophagitis cases.

One striking finding in our study is the high frequency of upper gastrointestinal tumors with endoscopic features of malignancy, observed in 17.2%. This figure is significantly higher than the 8.9% and 8.2% reported in studies from St. Paul’s Hospital 14 and Gondar 15 . Prevalence of esophageal cancer in our study is notably higher at 11.3%, compared with only 4.3% reported in studies from Gondar 15 and Addis Ababa 14 . Khat chewing and the associated high frequency of concomitant cigarette smoking and alcohol intake practiced in the region contribute to high incidence of cancers 21 . This notable discrepancy may also be attributed to regional differences in environmental and lifestyle factors, such as dietary habits and genetic predispositions, as well as variations in access to healthcare and screening practices, which could lead to increased detection of advanced malignancies in our population.

Another key finding is that 52.2% of patients with upper gastrointestinal tumors were aged 50 years or younger, with an average symptom duration of 6.9 months before undergoing endoscopy, indicating considerable diagnostic delays. The high prevalence of cancer among younger patients and prolonged symptom duration align with previous studies from Ethiopia 14 15 26 , underscoring the urgent need for timely referrals and better access to diagnostics.

Histologic findings also reveal a changing trend, with the proportion of esophageal adenocarcinoma among histologically confirmed cases rising from 11% three decades ago in Addis Ababa 17 to 17.5% among esophageal and 76% among gastroesophageal junction tumors in our current study. This suggests potential shifts in etiological patterns over time, consistent with observations from other regions of Ethiopia, including the high prevalence of GERD in recent years 16 25 .

The high rate of endoscopic findings indicating inflammation, such as gastritis and/or duodenitis (33.3%), including 14.5% among patients diagnosed with esophagitis, is also concerning. Combined with similarly high rates of histologic evidence of inflammation, these findings highlight the ongoing impact of *H. pylori* infection as a significant factor in upper gastrointestinal disorders despite efforts to improve its management 18 . Of the 425 documented biopsy results, approximately 22% showed lesions linked to *H. pylori* infection, including chronic nonspecific gastritis and chronic atrophic gastritis, some with intestinal metaplasia, a precursor lesion for gastric cancer 27 . These findings underscore the need for improved diagnostics, effective treatment protocols, and public health efforts to control *H. pylori* and reduce upper gastrointestinal disorders.

Besides the direct impact on patient care, the success of this outreach model has also facilitated capacity-building among local healthcare providers. Training provided to nurses in patient preparation, equipment handling, and post-procedure care has increased local procedure knowledge, resulting in improved procedure support. N addition, increased awareness about gastrointestinal disease burden has sparked interest among private and governmental institutions in expanding gastroenterology services in underserved areas, with some regional universities now considering specialized training programs in gastroenterology paving the way for sustainability of access to services.

### Strengths and limitations

The large sample size of over 2,000 patients enhances statistical power and provides valuable insights into the local context in the Jimma region. Combining endoscopic observations with histopathological diagnoses offers a comprehensive view of upper gastrointestinal disease patterns, including trends in GERD and malignancies. Comparisons with other regions help contextualize findings and highlight the importance of early detection, especially in patients with alarm symptoms.

However, this study has several limitations. Being based in a referral center may skew findings toward more severe cases, limiting generalizability to the broader Ethiopian population. GERD diagnosis, primarily based on clinical and endoscopic features without pH monitoring, may affect accuracy. In addition, not all diagnoses were confirmed by histopathology, reducing specificity for certain inflammatory conditions. Limited data on lifestyle factors and resource constraints affecting *H. pylori* testing may also impact the observed associations. All the other centers in Ethiopia shared similar limitations and used similar approaches.

## Conclusions

The weekend outreach endoscopy program has proven feasible, impactful, and sustainable in addressing the gastrointestinal healthcare gap in Southwest Ethiopia. The findings underscore a significant unmet need for endoscopy services in rural areas, where limited diagnostic access and delayed referrals contribute to high rates of advanced disease at diagnosis. This outreach model, in collaboration with local facilities, offers a practical approach to extending diagnostic reach in resource-limited settings. Expansion of similar services, along with targeted research into regional risk factors, holds the potential to improve gastrointestinal healthcare access, early disease detection, and long-term outcomes in underserved populations.
